# Botanical Extracts from Rosehip (*Rosa canina*), Willow Bark (Salix alba), and Nettle Leaf (*Urtica dioica*) Suppress IL-1**β**-Induced NF-*κ*B Activation in Canine Articular Chondrocytes

**DOI:** 10.1155/2012/509383

**Published:** 2012-03-14

**Authors:** Mehdi Shakibaei, David Allaway, Simone Nebrich, Ali Mobasheri

**Affiliations:** ^1^Musculoskeletal Research Group, Institute of Anatomy, Ludwig-Maximilian-University Munich, 80336 Munich, Germany; ^2^Nutrition and Metabolism Research Group, WALTHAM Centre for Pet Nutrition, Waltham on the Wolds, Melton Mowbray, Leicestershire LE14 4RT, UK; ^3^Musculoskeletal Research Group, Division of Veterinary Medicine, School of Veterinary Medicine and Science, Faculty of Medicine and Health Sciences, University of Nottingham, Sutton Bonington Campus, Sutton Bonington, Leicestershire LE12 5RD, UK

## Abstract

The aim of this study was to characterize the anti-inflammatory mode of action of botanical extracts from rosehip (*Rosa canina*), willow bark (*Salix alba*), and nettle leaf (*Urtica dioica*) in an *in vitro* model of primary canine articular chondrocytes. *Methods.* The biological effects of the botanical extracts were studied in chondrocytes treated with IL-1*β* for up to 72 h. Expression of collagen type II, cartilage-specific proteoglycan (CSPG), *β*1-integrin, SOX-9, COX-2, and MMP-9 and MMP-13 was examined by western blotting. *Results.* The botanical extracts suppressed IL-1*β*-induced NF-*κ*B activation by inhibition of I*κ*B*α* phosphorylation, I*κ*B*α* degradation, p65 phosphorylation, and p65 nuclear translocation. These events correlated with downregulation of NF-*κ*B targets including COX-2 and MMPs. The extracts also reversed the IL-1*β*-induced downregulation of collagen type II, CSPG, *β*1-integrin, and cartilage-specific transcription factor SOX-9 protein expression. In high-density cultures botanical extracts stimulated new cartilage formation even in the presence of IL-1*β*. *Conclusions.* Botanical extracts exerted anti-inflammatory and anabolic effects on chondrocytes. The observed reduction of IL-1*β*-induced NF-*κ*B activation suggests that further studies are warranted to demonstrate the effectiveness of plant extracts in the treatment of OA and other conditions in which NF-*κ*B plays pathophysiological roles.

## 1. Introduction

Osteoarthritis (OA) is a joint disease involving not only articular cartilage but also the synovial membrane, subchondral bone and periarticular soft tissues [1]. OA may occur following traumatic injury to the joint, subsequent to an infection of the joint or simply as a result of aging. The symptoms and signs characteristic of OA in the most frequently affected joints are heat, swelling, pain, stiffness and limited mobility. Other sequelae include osteophyte formation and joint malalignment. These manifestations are highly variable, depending on joint location and disease severity [2]. OA is grossly characterized by aberrant synthesis of extracellular matrix, gradual hypocellularity, eventual fragmentation and degradation of cartilage, new bone formation in the periarticular region (osteophytosis), decreased, then increased, subchondral bone density, and variable synovial inflammation [3]. In OA, mechanical stress initiates cartilage lesions by altering chondrocyte-matrix interaction and metabolic responses in the chondrocytes [4].

The interaction between chondrocytes and matrix proteins is mediated largely by the *β*1-integrin receptors [5]. This interaction plays a crucial role in regulating several biological phenomena, including cell morphology, gene expression, and cell survival. *β*1-integrins are transmembrane signal transduction receptors mediating cell-matrix interactions in cartilage [6]. An important signal transduction pathway activated by *β*1-integrin receptors is the MAPKinase pathway [7, 8]. Furthermore, disruption of cell matrix communication by inhibition of the MAPKinase pathway has been shown to lead to caspase-3 activation, cleavage of Poly(ADP)Ribose polymerase, and chondrocyte apoptosis [8].

There are initial increases in the amounts of water and proteoglycans associated with the observed transient chondrocyte proliferation of early OA. Proliferating chondrocytes appear in clusters and are accompanied by a change in cellular morphology and phenotype, indicating a hypertrophic differentiation process. At the molecular level, OA is characterized by loss of cartilage matrix components, particularly type II collagen and aggrecan due to an imbalance between extracellular matrix destruction and repair [9]. Although OA chondrocytes have increased expression of both anabolic and catabolic matrix genes [10], their catabolic ability is considered to dominate their anabolic capacity resulting in cartilage loss. As OA progresses from mild to severe, there is a decrease in transcription of collagen, failure to maintain the proteoglycan matrix, and reduced ability of the chondrocytes to regulate apoptosis [11]. In contrast, collagen type X, which is normally produced by terminally differentiated hypertrophic chondrocytes, has been demonstrated in surrounding chondrocyte clusters in OA cartilage [12]. Chondrocyte proliferation (cloning) is considered to be an attempt to repair and counteract cartilage degradation. However, disease progression and secondary inflammation indicate that this is generally unsuccessful. The short-lived hyperplasia (chondrocyte cloning) is followed by hypocellularity and apoptosis [13]. Catabolic events responsible for cartilage matrix degradation include (i) the release of catabolic cytokines such as IL-1*β*, IL-6, and TNF-*α* [14]; (ii) the production of matrix degrading enzymes such as matrix metalloproteinases (MMPs), mainly, stromelysin-1 (MMP-3) and collagenase-3 (MMP-13); (iii) reactive oxygen species (ROS) production by chondrocytes in OA [4, 14]. Imbalance between MMPs and tissue inhibitors of MMPs (TIMPs) occurs, resulting in active MMPs and consequent cartilage matrix degradation. However, IL-1*β* may also contribute to the depletion of cartilage matrix by decreasing synthesis of cartilage specific proteoglycans and collagen type II [4, 15]. 

The proinflammatory effects of IL-1*β* and TNF-*α* in OA are regulated by the transcription factor “nuclear transcription factor *κ*B” (NF-*κ*B) [16]. The subunits of NF-*κ*B (p65 and p50) are located in the cytoplasm as an inactive complex in association with an inhibiting I*κ*B*α* subunit. In response to phosphorylation, I*κ*B*α* dissociates from the complex and NF-*κ*B translocates to the cell nucleus and binds to target genes of NF-*κ*B [17]. NF-*κ*B might be also responsible for downregulation of the transcription factor SOX-9, which is involved in the regulation of genes for cartilage-specific extracellular matrix (ECM) proteins [18].

Nonsteroidal anti-inflammatory drugs (NSAIDs) are currently the most widely used anti-inflammatory drugs. However, they exhibit numerous undesired side effects and are only temporarily effective. Therefore, naturally occurring botanical extracts capable of inhibiting NF-*κ*B mediated catabolic activity may prove to be promising therapeutic agents for the treatment of OA [19]. Plant extracts with anti-inflammatory activity may also help to reduce the frequency of consumption and dosage of NSAIDs in arthritis patients. In recent years there has been a proliferation of research into botanical extracts with potential anti-inflammatory properties [20]. The most important factor that drives the interest in botanical extracts is the realization that inflammation plays a central role in the development of many chronic diseases in humans and companion animals. The use of herbal medicine is increasing among human arthritis patients in the United States and western Europe [21]. According to The Arthritis Foundation, almost 45% of patients in North America apply ointments or rubs for OA. A variety of topical and oral preparations are currently available. Many of these are traditional Chinese, Indian, or Korean herbal medicines, which have been used in the armamentarium of indigenous practitioners. The best-studied botanicals investigated to date include rosehip (*Rosa canina*) [22], *Tripterygium wilfordii* Hook F extract [23], Triptolide [24], Devil's Claw (*Harpagophytum procumbens*) [25], ginger (*Zingiber officinale Rosc.*) [26], and *Withania somnifera (ashwagandha) *[27]. In addition, conventional [28] and several systematic reviews [29] have examined published evidence for the effectiveness of botanical anti-inflammatory drugs. A few of these have specifically focused on the treatment of OA and chronic low back pain [30, 31]. 

The present study was designed to characterize the effects and mechanism of action of three botanical extracts; rosehip (*Rosa canina*), willow bark (*Salix alba*), and nettle leaf (*Urtica dioica*) in primary canine articular chondrocytes. Previous studies have reported on the anti-inflammatory activity of *Urtica dioica* [17, 30, 31]. In this study, we show that these botanical extracts exhibit a strong capacity for the inhibition of NF-*κ*B and its regulated gene products in chondrocytes *in vitro*.

## 2. Materials and Methods

### 2.1. Antibodies

Antibodies to collagen type II (AB746), *β*1-integrin (MAB1977), and cartilage-specific proteoglycan antibody (MAB2015) were purchased from Millipore (Schwalbach, Germany). Secondary antibodies were purchased from Dianova (Hamburg, Germany). Antibodies to *β*-actin (A5316) were from Sigma (Munich, Germany). Antibodies raised against MMP-9 (MAB911) and MMP-13 were purchased from R&D Systems (Abingdon, UK). Cyclo-oxygenase-2 (COX-2) (160-112) antibody was obtained from Cayman Chemical (Ann Arbor, MI, USA). Monoclonal anti-ERK antibody and polyclonal anti-Shc antibody were purchased from Becton Dickinson (Heidelberg, Germany). Antibodies to p65 (IMG-512), phospho-I*κ*B*α* (IMG-156A) and pan-I*κ*B*α* (IMG-127), were obtained from Biocarta (Hamburg, Germany). Antibodies to NF-*κ*B p65 (Rel A) and phospho-specific pS529 (100-401-266) were obtained from Rockland laboratories (Biomol, Hamburg, Germany). SOX-9 antibody was purchased from Acris Antibodies GmbH (Hiddenhausen, Germany). Peptide aldehydes and a specific proteosome inhibitor N-Ac-Leu-Leu-norleucinal (ALLN) were obtained from Boehringer Mannheim (Mannheim, Germany). The MTT assay was purchased from Sigma (Munich, Germany).

### 2.2. Culture Medium and Chemicals

Culture medium (Ham's F-12/Dulbecco's modified Eagle's medium (50/50) containing 10% fetal calf serum (FCS), 25 *μ*g/mL ascorbic acid, 50 IU/mL streptomycin, 50 IU/mL penicillin, 2.5 *μ*g/mL amphotericin B, essential amino acids and L-glutamine) was obtained from Seromed (Munich, Germany). Trypsin/EDTA (EC 3.4.21.4) was purchased from Sigma (Munich, Germany). Epon was obtained from Plano (Marburg, Germany). IL-1*β* was obtained from Strathman Biotech GmbH (Hannover, Germany).

### 2.3. Preparation of the Botanical Extracts

The botanical extracts from rosehip (*Rosa canina*), white willow bark (*Salix alba*) and nettle leaf (*Urtica dioica*) were supplied as powders from Shamanshop, Camden, NY, USA; catalogue numbers (202055-51_C, 202295-51_C, and 201865-51_C, resp.). The botanical extracts were prepared using chloroform, a commonly used solvent in the laboratory because it is relatively unreactive, miscible with most organic liquids, and conveniently volatile. Also, plant material is commonly extracted with chloroform for pharmaceutical processing. Chloroform solvent extractions were carried out by Puleva Biotech, Granada, Spain. Each botanical extract (~10 g) was packed in filter paper and loaded into the main chamber of a Soxhlet extraction unit. Chloroform (200 mL) was heated to reflux and incubated for 2 h. Following chloroform-evaporation under vacuum in a rotary evaporator (placed in a water bath at 60°C) the soluble botanical extract was dissolved in dimethyl sulfoxide (DMSO) at a stock concentration of 10 mg/mL and stored in aliquots at −80°C. The final concentration of DMSO did not, in any case, exceed 0.1%. Further dilutions were made in cell culture medium to achieve the final working concentrations.

### 2.4. Chondrocyte Isolation and Culture

Primary canine articular chondrocytes were isolated from the joints of client-owned dogs undergoing orthopaedic surgery at the Clinic of Veterinary Surgery, Ludwig-Maximilian-University Munich, Germany. Fully informed owner consent was obtained and the Ethical Review Committees of WALTHAM, Ludwig-Maximilian-University, and the University of Nottingham approved the project. Cartilage explants were sliced and digested primarily with 1% pronase for 2 h at 37°C and subsequently with 0.2% collagenase for 4 h at 37°C. Isolated chondrocytes were maintained in culture medium at a density of 0.1 × 10^6^ cells/mL in Petri dishes in monolayer culture and on glass plates for a period of 24 h at 37°C with 5% CO_2_.

### 2.5. Experimental Design

Serum-starved chondrocytes (passage two, cultivated in 3% FCS) were treated with the botanical extracts (10 *μ*g/mL) alone for 24 h (pretreatment) and then cotreated with a combination of botanical extracts (10 *μ*g/mL) and IL-1*β* (10 ng/mL) for a further 48 h in monolayer cultures. Chondrocytes treated with the botanical extracts alone over the entire period served as treatments and those treated with IL-1*β* were used as “inflammatory” controls. In addition, untreated chondrocytes (i.e., cells only exposed to serum-starved medium) served as untreated controls. For investigation of NF-*κ*B translocation and I*κ*B*α* phosphorylation, chondrocytes were treated either with IL-1*β* (10 ng/mL) or cotreated with a combination of botanical extracts (10 *μ*g/mL) and IL-1*β* (10 ng/mL) for 0, 15, 30, and 60 min and nuclear/cytoplasmic extracts were prepared.

### 2.6. MTT Assay

Chondrocytes were seeded in 96-well plates with 5000 cells/well and incubated overnight in culture medium containing 10% FCS. Positive control cells were left untreated or were treated with the compounds alone. Negative controls were cells treated with IL-1*β* alone. Additionally, chondrocytes were incubated only with the same quantity of DMSO in serum starved medium as in working solutions (without the botanical extracts). For every control and experimental treatment, three wells were used. For measurements after 0, 24, 48, and 72 h, the medium (with or without botanical extracts) was replaced with serum-starved medium and MTT (10 *μ*L) was added. After incubation for 4 h at 37°C MTT solubilization solution was added and cells were incubated at 37°C until MTT formazan crystals were completely dissolved. Absorbance was measured at a wavelength of 550 nm with a spectrophotometer.

### 2.7. Immunofluorescence Microscopy

Cells were cultivated on glass plates and incubated for 24 h. The cells were then washed three times and preincubated for 1 h with serum-starved medium before stimulation with 10 ng/mL IL-1*β* or 10 *μ*g/mL botanical extracts alone or cotreated with 10 *μ*g/mL botanical extracts and 10 ng/mL IL-1*β* for 30 min in serum-starved (3% FCS) medium. Cells on the glass plates were washed three-times in Hanks solution before methanol fixation for 10 min at ambient temperature (AT), and rinsing with phosphate-buffered saline (PBS). Cell and nuclear membranes of chondrocytes were permeabilized by treatment with 0.1% Triton X-100 for 1 min on ice. Cells were washed with bovine serum albumin (BSA) for 10 min at AT, rinsed with PBS, and incubated with primary antibodies (p65, phospho-p65, 1 : 30 in PBS). They were gently washed several times with PBS before incubation with secondary antibody (goat-anti-rabbit immunoglobulin conjugated with FITC, diluted 1 : 50 in PBS). Glass plates were finally washed three-times with PBS, covered with fluoromount mountant, and examined under a light microscope (Axiophot 100, Zeiss, Germany).

### 2.8. Isolation of Nuclear and Cytoplasmic Chondrocyte Extracts

Chondrocytes were trypsinized and washed twice in ice-cold PBS (1 mL). The supernatant was removed and cell pellets were resuspended in hypotonic lysis buffer (400 *μ*L) containing protease inhibitors. After incubation on ice for 15 min, 10% NP-40 (12.5 *μ*L) was added and the cell suspension was vigorously mixed for 15 sec. The extracts were centrifuged for 1.5 min. The supernatants (cytoplasmic extracts) were frozen at −70°C. Ice-cold nuclear extraction buffer (25 *μ*L) was added to the pellets and incubated for 30 min with intermittent mixing. Extracts were centrifuged and the supernatant (nuclear extracts) transferred to pre-chilled tubes for storage at −70°C.

### 2.9. High-Density Cultures

The high density mass culture was performed on a steel grid bridge as previously described [5]. Briefly, a cellulose filter was placed on the bridge onto which a cell suspension (8 *μ*L), containing approximately 1 million cells, was placed. Culture medium was in contact with the filter and the cells were maintained at the filter-medium interface through diffusion. After one day in culture, cells formed a three-dimensional pellet on the filter. Culture medium was changed every three days.

### 2.10. Transmission Electron Microscopy (TEM)

Cells were fixed for 1 h with Karnovsky's fixative (paraformaldehyde-glutaraldehyde) followed by postfixation in 1% OsO_4_ solution (0.1 M phosphate buffer), as previously described [32]. Monolayer cell pellets were rinsed and dehydrated in an ascending alcohol series before being embedded in Epon and cut on a Reichert-Jung Ultracut E (Darmstadt, Germany). Ultrathin sections were contrasted with 2% uranyl acetate/lead citrate. A transmission electron microscope (TEM 10, Zeiss, Jena, Germany) was used to examine the cultures.

### 2.11. Western Blot Analysis

Chondrocyte monolayers were washed three times with Hank's balanced salt solution (HBSS) and whole cell proteins were extracted by incubation with lysis buffer (50 mM Tris/HCl, pH 7.2, 150 mM NaCl, l% (v/v) Triton X-100, 1 mM sodium orthovanadate, 50 mM sodium pyrophosphate, 100 mM sodium fluoride, 0.01% (v/v) aprotinin, 4 *μ*g/mL pepstatin A, 10 *μ*g/mL leupeptin, 1 mM PMSF) on ice for 30 min, and cell debris was removed by centrifugation. Supernatants were stored at −70°C. Total protein concentration of whole cell, nuclear and cytoplasmic extracts was determined according to the bicinchoninic acid system (Uptima, Interchim, Montlucon, France) using BSA as a standard. After adjusting the equal amounts (50 *μ*g of protein per lane) of total protein, proteins were separated by SDS-PAGE (5, 7.5% gels) under reducing conditions. The separated proteins were transferred onto nitrocellulose membranes. Membranes were preincubated in blocking buffer (5% (w/v) skimmed milk powder in PBS/0.1% Tween-20) for 30 min and incubated with primary antibodies (1 h, AT). Membranes were washed three times with blocking buffer and incubated with alkaline phosphatase conjugated secondary antibodies for 30 min. They were finally washed three times in 0.1 M Tris pH 9.5 containing 0.05 M MgCl_2_ and 0.1 M NaCl. Nitro blue tetrazolium and 5-bromo-4-chloro-3-indoylphosphate (p-toluidine salt; Pierce, Rockford, IL, USA) were used as substrates to reveal alkaline phosphatase-conjugated specific antigen-antibody complexes. 

### 2.12. Statistical Analysis

The results are expressed as the means ± SD of a representative experiment performed in triplicate. The means were compared using student's *t*-test assuming equal variances and *P* < 0.05 was considered statistically significant.

## 3. Results

This *in vitro *study was undertaken to investigate the anti-inflammatory effect of three botanical extracts on the signaling pathway leading to the activation of the transcription factor NF-*κ*B and a selection of its target gene products, namely, proteins important to chondrocyte function. Chondrocytes treated with botanical extracts (10 *μ*g/mL) showed no signs of cytotoxicity at the light and electron microscopic (ultrastructural) levels. IL-1*β* was used to examine the effect of botanical extracts on the NF-*κ*B activation pathway, because the pathway activated by this cytokine is relatively well understood. 

### 3.1. Botanical Extracts Suppress IL-1*β*-Induced Chondrocyte Cytotoxicity

**Figure 1:**
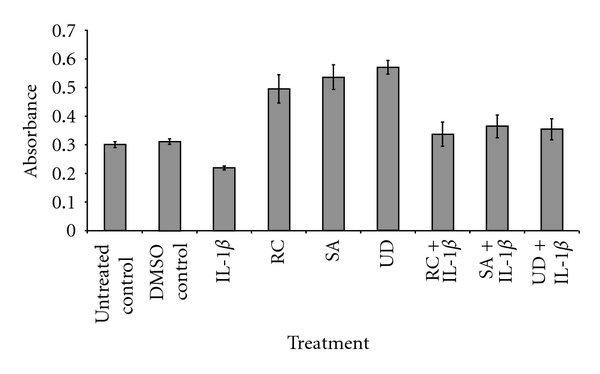
Effect of botanical extracts and IL-1*β* on the proliferation of chondrocytes *in vitro*. Serum-starved chondrocytes were exposed to IL-1*β* (10 ng/mL) for 48 h, botanical extracts (10 *μ*g/mL) for 72 h, cotreated first with botanical extracts (10 *μ*g/mL each) for 24 h, and then with IL-1*β* (10 ng/mL) for 48 h, treated with DMSO (as control) for 72 h or left untreated for 72 h. Cell viability was examined by MTT assay. The MTT assay is a spectrophotometric measurement of the cell viability as a function of the mitochondrial activity. This assay was performed in triplicate and the results are provided as mean values with standard deviations from three independent experiments. Treatments: Untreated control; IL-1*β*; RC (*Rosa canina*); SA (*Salix alba*); UD (*Urtica dioica*).

To test IL-1*β*-inhibited chondrocyte proliferation an MTT assay was performed to study the effects of botanical extracts on the viability and proliferation of chondrocytes treated with or without IL-1*β*. The MTT assay is based on the ability of living cells to reduce the MTT salt, whereas dead cells or those with impaired mitochondrial activity are unable to do so. Chondrocytes were cultured in a 96-well plate and treated with IL-1*β*, botanical extracts, and botanical extracts then treated with IL-1*β* for the indicated times. The viability and proliferation of the chondrocytes cultivated only in the presence of IL-1*β* was significantly lower compared to those of chondrocytes treated with botanical extracts, botanical extracts and IL-1*β*, or left untreated (Figure 1). The results showed a positive effect of three botanical extracts with regard to cell viability and proliferation on inhibiting IL-1*β*-induced cytotoxicity on chondrocytes.

### 3.2. Botanical Extracts Block IL-1*β*-Induced Cellular/Ultrastructural Changes and Apoptosis in Chondrocytes

**Figure 2:**
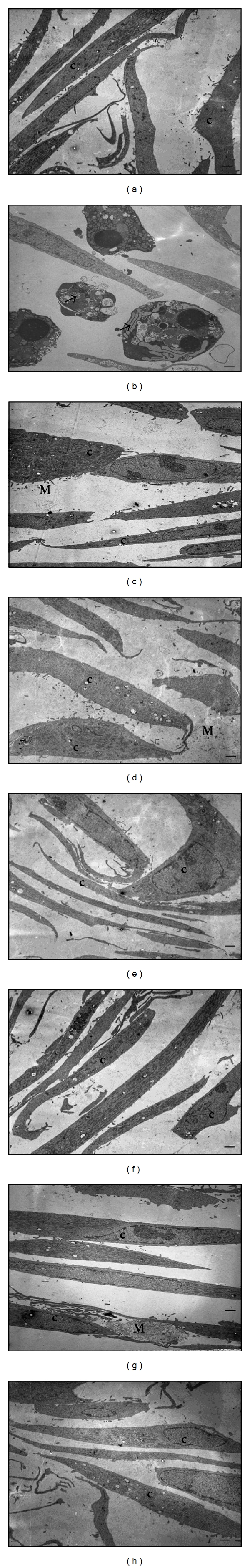
Effect of botanical extracts on IL-1*β*-induced cell degradation and apoptosis. Serum-starved chondrocytes were either left untreated, (a) exposed to IL-1*β* (10 ng/mL) alone (b), or to botanical extracts alone (f–h) for 1, 12, 24, 48, and 72 h or pretreated for 24 h with botanical extracts (10 *μ*g/mL) before being cotreated with IL-1*β* (10 ng/mL) and botanical extracts (10 *μ*g/ml) (c–e) and evaluated with TEM. Chondrocytes treated with IL-1*β* (10 ng/mL) exhibited characteristic features of degeneration: annular chromatin condensation at the nuclear envelope of chondrocytes, swelling of mitochondria, and rough ER in a time-dependent manner (b). Chondrocytes that were pretreated with botanical extracts and then cotreated with IL-1*β* and botanical extracts (panels c–e) showed less severe cell degeneration at the ultrastructural level. In control cultures (a) and treated with botanical extracts alone (panels f–h) showed no ultrastructural changes. A–M: ×5000; Bar = 1 *μ*m. Treatments: *Rosa canina* + IL-1*β*, panel (c); *Salix alba* + IL-1*β*, panel (d); *Urtica dioica* + IL-1*β*, panel (e); *Rosa canina* without IL-1*β*, panel (f); *Salix alba* without IL-1*β*, panel (g); *Urtica dioica* without IL-1*β*, panel (h).

Control monolayer chondrocytes after 24 (not shown), 48 (Figure 2(a)), and 72 h (not shown) showed a typical flattened shape with small cytoplasmic processes, a large, mostly euchromatic nucleus with nucleoli and a well-structured cytoplasm. IL-1*β*-treatment of chondrocyte monolayer cultures for 24 (data not shown) and 48 h (Figure 2(b)) lead to degenerative changes such as multiple vacuoles, swelling of rough ER, clustering of swollen mitochondria, and degeneration of other cell organelles. After longer incubation periods (72 h) (data not shown) more severe features of cellular degeneration were seen in response to IL-1*β* treatment. These included areas of condensed heterochromatin in the cell nuclei and multiple cytoplasmic vacuoles. The flattened monolayer chondrocytes became increasingly rounded and apoptotic (Figure 2(b)). Chondrocytes pretreated with any of the botanical extracts (10 *μ*g/mL) (24 h) and then cotreated with IL-1*β* and the same botanical extracts (10 *μ*g/mL) for 48 h showed less severe cellular degeneration on the ultrastructural level (Figures 2(c)–2(e)). The chondrocytes remained a flattened shape with numerous microvilli-like cytoplasmic processes. Chondrocytes treated with botanical extracts alone (each at 10 *μ*g/mL) showed no signs of cytotoxic effects on the viability of cells at the light microscopic and ultrastructural levels (Figures 2(f)–2(h)). Taken together, these results indicate that all three botanical extracts have antiapoptotic effects and counteract IL-1*β*-induced apoptosis in chondrocytes. 

### 3.3. Botanical Extracts Inhibit IL-1*β*-Induced Downregulation of Extracellular Matrix and Signaling Proteins in Chondrocytes

**Figure 3:**
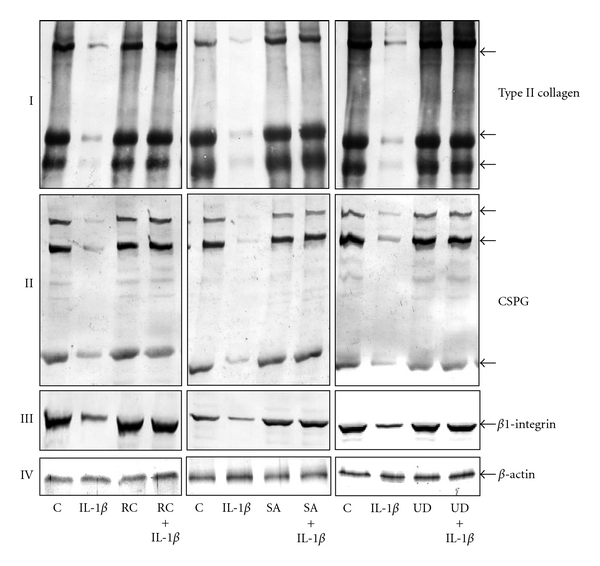
Effects of botanical extracts on IL-1*β*-induced downregulation of extracellular matrix and signaling proteins in chondrocytes. Serum-starved chondrocytes (0.1 × 10^6^ cells/mL) were cultured for 24 h and then treated with 10 ng/mL IL-1*β* for 48 h, botanical extracts (each 10 *μ*g/ml) for 72 h, or pretreated with botanical extracts (10 *μ*g/mL each) for 24 h and then cotreated with 10 ng/mL IL-1*β* for 48 h or left untreated and evaluated after 72 h. Western blot analysis revealed down-regulation of collagen type II (I), CSPG (II) and *β*1-integrin (III) in chondrocytes by IL-1*β*. Co-treatment of chondrocytes preincubated with botanical extracts and IL-1*β* suppressed the IL-1*β*-induced inhibition of collagen type II, CSPG and *β*1-integrin (I, II, III). In untreated and in botanical extracts alone treated control cultures, expression of collagen type II, CSPG and *β*1-integrin were equally strong in chondrocytes (I–III). Expression of *β*-actin was not affected by IL-1*β* and/or botanical extracts (IV). Data shown are representative of three independent experiments. Treatments: C (untreated control); IL-1*β*; RC (*Rosa canina*); SA (*Salix alba*); UD (*Urtica dioica*).

Serum-starved chondrocytes were treated with IL-1*β* (10 ng/mL) alone or were preincubated with three different botanical extracts (10 *μ*g/mL each) for 24 h and then cotreated with IL-1*β* (10 ng/mL) for 24, 48, and 72 h. As shown in Figure 3, chondrocytes stimulated with IL-1*β* alone showed downregulation of synthesis of collagen type II (Figure 3(I)), cartilage-specific proteoglycan (CSPG) (Figure 3(II)), and *β*1-integrin (Figure 3(III)). In contrast to chondrocytes stimulated with IL-1*β* alone, pretreatment with all botanical extracts resulted in a significant up-regulation of synthesis of collagen type II (Figure 3(I)), CSPG, (Figure 3(II)) and *β*1-integrin (Figure 3(III)). In untreated and in positive control cultures, expression of collagen type II, CSPG, and *β*1-integrin were equally strong in chondrocytes (Figures 3(I)–3(III)). Synthesis of the housekeeping protein *β*-actin remained unaffected in chondrocytes exposed to botanicals (Figure 3(IV)). 

### 3.4. Botanical Extracts Inhibit IL-1*β*-Induced Upregulation of NF-*κ*B-Dependent ProInflammatory Enzymes and Matrix Degrading Gene Products in Chondrocytes

**Figure 4:**
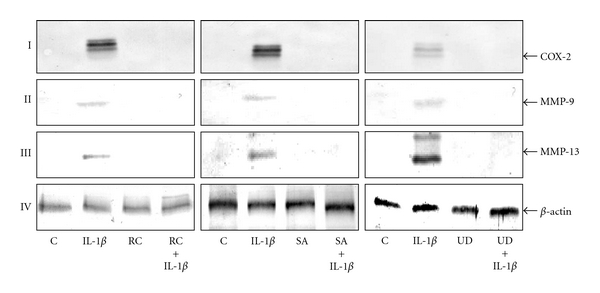
Effects of botanical extracts on IL-1*β*-induced upregulation of proinflammatory enzymes in chondrocytes. Serum-starved chondrocytes (0.1 × 10^6^ cells/mL) were cultured for 24 h and then treated with 10 ng/mL IL-1*β* for 48 h, botanical extracts (each 10 *μ*g/mL) for 72 h, or pretreated with botanical extracts (10 *μ*g/mL each) for 24 h and then cotreated with 10 ng/mL IL-1*β* for 48 h or left untreated and evaluated after 72 h. IL-1*β*-stimulation leads to an increase in synthesis of COX-2, MMP-9, and MMP-13 (I, II, III). However, COX-2, MMP-9, and MMP-13 upregulation was blocked in chondrocytes pre-incubated with botanical extracts (10 *μ*g/mL each) for 24 h and then co-treated with IL-1*β* (10 ng/mL) for 48 h (I, II, III). In untreated and in botanical extracts alone treated control cultures, expression of COX-2-, MMP-9 and MMP-13 were not seen in chondrocytes (I–III). Expression of the housekeeping gene *β*-actin was not affected by treatment with IL-1*β* and/or botanical extracts (IV). Data shown are representative of three independent experiments. Treatments: C (untreated control); IL-1*β*; RC (*Rosa canina*); SA (*Salix alba*); UD (*Urtica dioica*).

IL-1*β* stimulation activates COX-2 and MMPs expression in chondrocytes [33]. To investigate whether the three botanical extracts were able to inhibit IL-1*β*-induced expression of these proteins, the following experiment was performed. Serum-starved chondrocytes were exposed to IL-1*β* (10 ng/mL) alone or were preincubated with three different botanical extracts (10 *μ*g/mL each) for 24 hours and then co-treated with IL-1*β* (10 ng/mL) for 24, 48 and 72 h. The whole cell extracts were prepared and analyzed by western blotting for the presence of COX-2, MMP-9 and MMP-13 (Figures 4(I)–4(III)). As shown in Figure 4, chondrocytes showed up-regulation of synthesis of COX-2 (Figure 4(I)), MMP-9 (Figure 4(II)) and MMP-13 (Figure 4(III)) in response to IL-1*β* (10 ng/mL). In contrast to chondrocytes stimulated with IL-1*β* alone, pre-treatment with all botanical extracts and co-treatment with IL-1*β* led to a decrease in COX-2, MMP-9 and MMP-13 expression (Figures 4(I)–4(III)). In untreated and positive control cultures, expression of COX-2, MMP-9, and MMP-13 was not detectable in chondrocytes (Figures 4(I)–4(III)). Synthesis of the housekeeping protein *β*-actin remained unaffected (Figure 4(IV)). 

### 3.5. Botanical Extracts Inhibit the IL-1*β*-Induced Downregulation of Adaptor Protein Shc, Signaling Protein P-ERK1/2, and Cartilage-Specific Transcription Factor SOX-9 Expression in Chondrocytes

**Figure 5:**
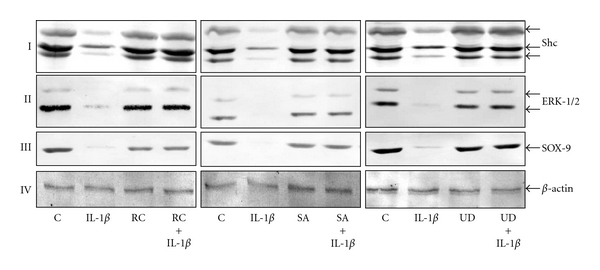
Effect of botanical extracts on signaling proteins and cartilage-specific transcription factor SOX-9 in chondrocytes. Serum-starved chondrocytes (0.1 × 10^6^ cells/mL) were cultured for 24 h and then treated with 10 ng/mL IL-1*β* for 48 h, botanical extracts (each 10 *μ*g/mL) for 72 h, or pretreated with botanical extracts (10 *μ*g/mL each) for 24 h and then cotreated with 10 ng/mL IL-1*β* for 48 h or left untreated and evaluated after 72 h. Results of western blot analysis revealed down-regulation of Shc (I), ERK1/2 (II), and SOX-9 (III) in chondrocytes with IL-1*β*. Cotreatment of chondrocytes preincubated with botanical extracts and IL-1*β* relieved the IL-1*β*-induced inhibition of Shc (I), ERK1/2 (II), and SOX-9 (III). In untreated and in positive control cultures, expression of Shc, ERK1/2, and SOX-9 were equally strong in chondrocytes (I–III). Expression of *β*-actin was not affected by IL-1*β* and/or botanical extracts (IV). Data shown are representative of three independent experiments. Treatments: C (untreated control); IL-1*β*; RC (*Rosa canina*); SA (*Salix alba*); UD (*Urtica dioica*).

The MAPKinase pathway plays an important role in chondrocyte differentiation and stimulates the chondrogenic factor SOX-9 in chondrocytes [7, 8]. SOX-9 is a transcription factor that controls the expression of chondrocyte-specific ECM protein genes and plays a pivotal role in chondrocyte differentiation, thus it was selected for this study. Additionally, the MAPKinase signaling pathway, the adaptor protein Shc and the extracellular regulated kinase (Erk1/2) were evaluated. To test the hypothesis that botanical extracts are able to stimulate SOX-9 production in chondrocytes, monolayer cultures were either left untreated or treated with IL-1*β* or botanical extracts alone or were pretreated with botanical extracts (10 *μ*g/mL) for 24 h and then stimulated with IL-1*β* for 24 h. The cell lysates were analyzed by immunoblotting. In untreated and in positive control cultures, expression of Shc, ERK1/2, and SOX-9 were equally strong in chondrocytes (Figures 5(I)–5(III)). The results demonstrated that treatment with the three botanical extracts inhibited the IL-1*β*-induced decrease in Shc, ERK1/2 and SOX-9 expression (Figures 5(I)–5(III)). Data shown are representative of three independent experiments. Synthesis of the housekeeping protein *β*-actin remained unaffected (Figure 5(IV)). 

### 3.6. Botanical Extracts Inhibit IL-1*β*-Induced NF-*κ*B Activation in Chondrocytes

**Figure 6:**
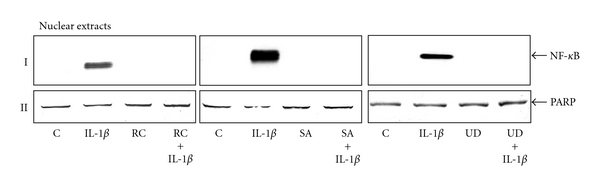
Botanical extracts block the IL-1*β*-induced phosphorylation and nuclear translocation of p65 in chondrocytes. Western blot analysis of IL-1*β*-treated nuclear extracts. Serum-starved chondrocytes (0.1 × 10^6^ cells/mL) were pretreated with botanical extracts (10 *μ*g/ML each) for 4 hours followed by cotreatment with 10 ng/mL IL-1*β* and botanical extracts for 1 h. Some chondrocyte cultures remained either untreated or were treated with 10 *μ*g/mL botanical extracts (each alone) or with 10 ng/mL IL-1*β* alone for 1 h. Nuclear extracts were probed for phospho p65, (I) by western blot analysis using antibodies to p65, phospho-specific p65, and PARP (II, control). Treatment of chondrocytes with IL-1*β* (10 ng/mL) revealed a clear increase in expression of phospho-p65 in the nuclear extracts (I). Co-treatment of chondrocytes with botanical extracts (all three) completely abolished the IL-1*β*-dependent activation of phospho p65 in the nucleus (I). Synthesis of PARP remained unaffected in nuclear extracts (II). Data shown are representative of three independent experiments. Treatments: C (untreated control); IL-1*β*; RC (*Rosa canina*); SA (*Salix alba*); UD (*Urtica dioica*).

To examine if botanical extracts block the IL-1*β*-induced activation of NF-*κ*B, nuclear protein extracts from serum-starved chondrocytes were probed for the phosphorylated form of p65 NF-*κ*B-subunit after pretreatment with botanical extracts (10 *μ*g/mL each) for 4 hours followed by cotreatment with 10 ng/mL IL-1*β* and botanical extracts for 1 h. Some chondrocyte cultures remained either untreated or were treated with 10 *μ*g/mL botanical extracts (each alone) or with 10 ng/mL IL-1*β* alone for 1 h (Figure 6(I)). Results indicate that botanical extracts inhibited IL-1*β*-induced NF-*κ*B activation (Figure 6(I)). The synthesis of the PARP protein remained unaffected (Figure 6(II)). 

### 3.7. Botanical Extracts Inhibit IL-1*β*-Stimulated Nuclear-Translocation of NF-*κ*B in Chondrocytes

**Figure 7:**
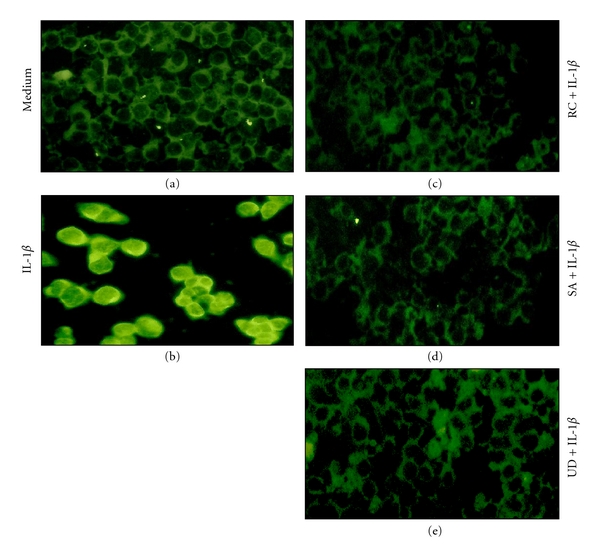
Botanical extracts inhibit IL-1*β*-induced nuclear translocation of NF-*κ*B in chondrocytes. Chondrocyte cultures either served as controls (a) or were treated with IL-1*β* alone for 10 min (b) or cotreated with botanical extracts for 10 min and then cotreated with IL-1*β* for 1 h (c–e) before immunolabeling with NF-*κ*B antibodies and FITC-coupled secondary antibodies. In control cells anti-NF-*κ*B labeling was restricted to the cytoplasm (a). Cells treated with IL-1*β* alone revealed nuclear translocation of NF-*κ*B (b) that was partly inhibited by cotreatment with botanical extracts (c–e). (a–e): ×160. Data shown are representative of three independent experiments.

Immunofluorescence microscopy was employed to reveal translocation of phosphorylated NF-*κ*B from the chondrocyte cytoplasm to the nucleus in response to IL-1*β*. Chondrocytes remained either unstimulated (Figure 7(a)) or were treated with 10 *μ*g/mL botanical extracts (each alone) or with 10 ng/mL IL-1*β* alone for 10 min (Figure 7(b)) or were cotreated with 10 *μ*g/mL botanical extracts (each alone) 10 min and then 10 ng/mL IL-1*β* for 1h (Figures 7(c)–7(e)) before indirect immunolabeling with anti-NF-*κ*B antibody. Control chondrocytes and chondrocytes treated with the botanical extracts alone (not shown) showed only cytoplasmic labeling of NF-*κ*B (Figure 7(a)). IL-1*β*-stimulated cells revealed clear and intensive cytoplasmic and nuclear staining for NF-*κ*B (Figure 7(b)). Cotreatment of chondrocytes with botanicals and IL-1*β* resulted in inhibition of nuclear transition of activated phosphor-p65 and decreased cytoplasmic staining for this protein and showed a decrease in activation of NF-*κ*B (Figures 7(c)–7(e)). These immunomorphological findings were consistent with the NF-*κ*B inhibition observed by western blotting. 

### 3.8. Botanical Extracts Inhibit IL-1*β*-Induced I*κ*B*α* Degradation in Chondrocytes

**Figure 8:**
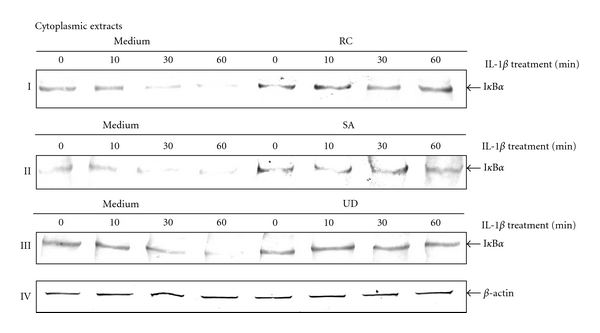
Effects of botanical extracts on the kinetics of I*κ*B*α* by IL-1*β* in chondrocytes. Effect of botanical extracts on IL-1*β*-induced degradation of I*κ*B*α*. Western blot analysis with IL-1*β*-treated cytoplasmic extracts. Serum-starved chondrocytes (0.1 × 10^6^ cells/mL) were treated with IL-1*β* (10 ng/mL) for 0, 10, 30 and 60 min. Other cultures were initially treated with botanical extracts (10 *μ*g/mL) for 4 h and then cotreated with IL-1*β* (10 ng/mL) for the indicated times or left untreated (medium controls). The cytoplasmic extracts were prepared, fractionated on SDS-PAGE, and electroblotted onto nitrocellulose membranes. Western blot analysis was performed with anti-I*κ*Ba and anti-*β*-actin (control). IL-1*β* caused I*κ*B*α* degradation in cultures as early as 10 min after treatment. In cotreated chondrocytes with each botanical extracts the degradation of I*κ*B*α* was not observed (I–III). Synthesis of *β*-actin remained unaffected (IV). Data shown are representative of three independent experiments. Treatments: medium (controls); IL-1*β*; RC (*Rosa canina*); SA (*Salix alba*); UD (*Urtica dioica*).

In this study, botanical extracts inhibited IL-1*β*-induced activation of NF-*κ*B and its translocation to the chondrocyte nucleus. An important prerequisite for the activation of NF-*κ*B is the phosphorylation and degradation of I*κ*B*α*, the natural blocker of NF-*κ*B [34]. To examine whether inhibition of IL-1*β*-induced NF-*κ*B activation occurs through inhibition of I*κ*B*α* degradation, some chondrocyte cultures were treated with IL-1*β* (10 ng/mL) for the indicated times (Figures 8(I)–8(III)) and other chondrocyte cultures were first treated with three botanical extracts (10 *μ*g/mL each) for 4 h followed by co-treatment with IL-1*β* (10 ng/mL) for the indicated time periods. IL-1*β* could not induce I*κ*B*α* degradation in chondrocytes when co-treated with botanical extracts (Figures 8(I)–8(III)). Considering, IL-1*β*-induced I*κ*B*α* degradation in untreated cultures is an indicator of NF-*κ*B activation, the results suggest that the botanical extracts block IL-1*β*-induced I*κ*B*α* degradation.

### 3.9. Botanical Extracts Inhibit IL-1*β*-Dependent I*κ*B*α* Phosphorylation in Chondrocytes

**Figure 9:**
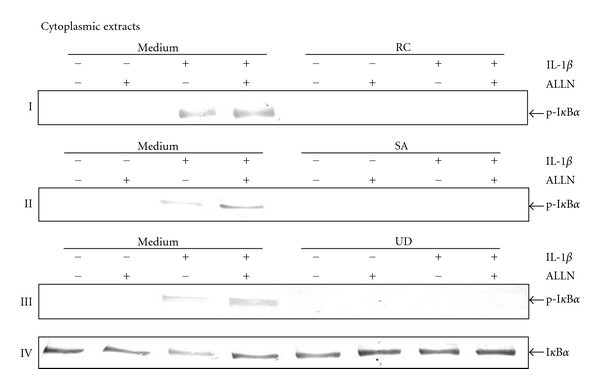
Effect of botanical extracts on the phosphorylation of I*κ*B*α* by IL-1*β* in chondrocytes. Western blot analysis with IL-1*β*-treated cytoplasmic extracts. Serum-starved chondrocytes (0.1 × 10^6^ cells/mL) were pretreated with ALLN (100 *μ*g/mL) for 30 min and cotreated with botanical extracts (each 10 *μ*g/mL) for 4 h and stimulated with IL-1*β* (10 ng/mL) for the final 1 h. The cytoplasmic extracts were prepared, fractionated on SDS-PAGE, and electroblotted onto nitrocellulose membranes. Western blot analysis was performed using anti-I*κ*B*α* and p-I*κ*B*α* antibodies. Treatment of chondrocytes with IL-1*β* (10 ng/mL) revealed an increase in the phosphorylated I*κ*B*α*-form in cytoplasmic extracts. In the presence of the inhibitor phosphorylation of I*κ*B*α* was significantly increased (I–III). Phosphorylation of I*κ*B*α* was inhibited in chondrocytes cotreated with botanical extracts in the presence or absence of the inhibitor (I–III). Data shown are representative of three independent experiments. Treatments: medium (controls); IL-1*β*; RC (*Rosa canina*); SA (*Salix alba*); UD (*Urtica dioica*).

To determine if the botanical extracts are able to inhibit the IL-1*β*-induced phosphorylation of I*κ*B*α*, serum-starved chondrocytes were treated with IL-1*β* for 1h and examined by western blot analysis using an antibody that recognizes the phosphorylated form of I*κ*B*α*. It is known that phosphorylation of I*κ*B*α* leads to its degradation [16], and that the phosphorylation and degradation of I*κ*B*α* are inhibited by a specific proteosome inhibitor N-Ac-Leu-Leu-norleucinal (ALLN) [35]. As shown in Figures 9(I)–9(III), IL-1*β* was still able to phosphorylate some I*κ*B*α* in cells pretreated with the inhibitor and I*κ*B*α* phosphorylation was significantly higher compared to control cells. Interestingly, all botanical extracts were able to inhibit the phosphorylation of I*κ*B*α* induced by IL-1*β* in the presence or absence of the inhibitor. 

### 3.10. Botanical Extracts Inhibit IL-1*β*-Induced Effects in a 3-Dimensional (High-Density) Culture Model of Chondrocytes

**Figure 10:**
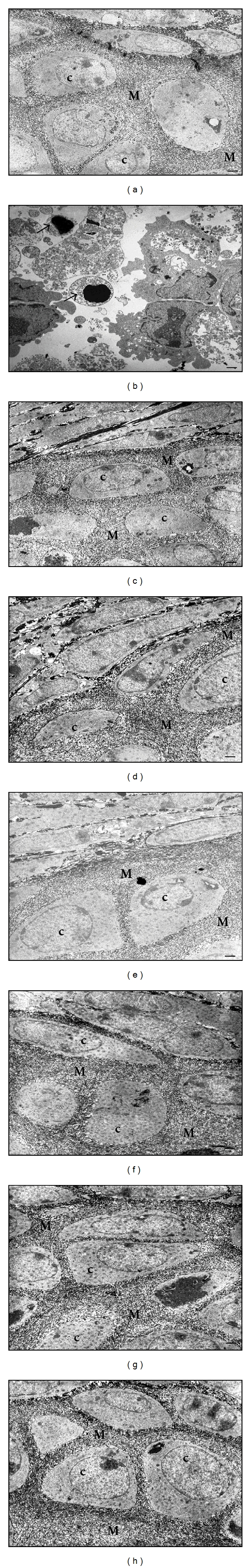
Cultivation of chondrocytes in a 3-dimensional culture system (High-density cultures) *in vitro in the presence of botanical extracts*. Chondrocytes were treated with botanical extracts (10 *μ*g/mL) or IL-1*β* (10 ng/mL) alone for 24 h before being treated with IL-1*β* (10 ng/mL) for 7 days in high-density cultures. Control cultures of chondrocytes showed well-developed cartilage nodules (a). Treatment with IL-1*β* resulted in cell destruction after 7 days (b). After the cotreatment of high-density cultures with botanical extracts and IL-1*β* (panels c–e) or in the absence of IL-1*β* (panels f–h) the chondrocytes exhibited well-developed cartilage nodules. ×4000; bars: 1 *μ*m. Treatments: *Rosa canina* + IL-1*β*, panel (c); *Salix alba* + IL-1*β*, panel (d); *Urtica dioica* + IL-1*β*, panel (e); *Rosa canina* without IL-1*β*, panel (f); *Salix alba* without IL-1*β*, panel (g); *Urtica dioica* without IL-1*β*, panel (h).

To test whether chondrocytes from monolayer cultures with or without IL-1*β* and/or botanicals were able to produce cartilage-specific ECM and cartilage, high-density cultures were prepared from chondrocytes in monolayer culture. These consisted of untreated control cells and cells treated with botanical extracts (10 *μ*g/mL) or IL-1*β* (10 ng/mL) alone for 24 h before being treated with IL-1*β* (10 ng/mL) and cultivated for 7 days under identical conditions. As shown in Figure 10, control cultures of chondrocytes formed blastema-like nodules and made tight contacts. They exhibited round to oval shapes, large euchromatic nuclei, free cytoplasmic ribosomes, mitochondria and endoplasmic reticulum (ER), as well as vacuoles. The cells appeared as viable chondrocytes exhibiting characteristic morphological features and formed a regular fibrillar extracellular matrix (Figure 10(a)). In contrast, chondrocytes underwent apoptosis when treated with IL-1*β* (10 ng/mL) for 7 days (Figure 10(b)). Chondrocytes co-treated with botanical extracts and IL-1*β* (each 10 *μ*g/mL) showed well-developed cartilage nodules (Figures 10(c)–10(e)). Pre-treatment with botanical extracts (each 10 *μ*g/mL) alone resulted in well-developed cartilage nodules with viable cells and organized organelles; the cells formed a dense and regular ECM (Figures 10(f)–10(h)). 

## 4. Discussion

**Figure 11:**
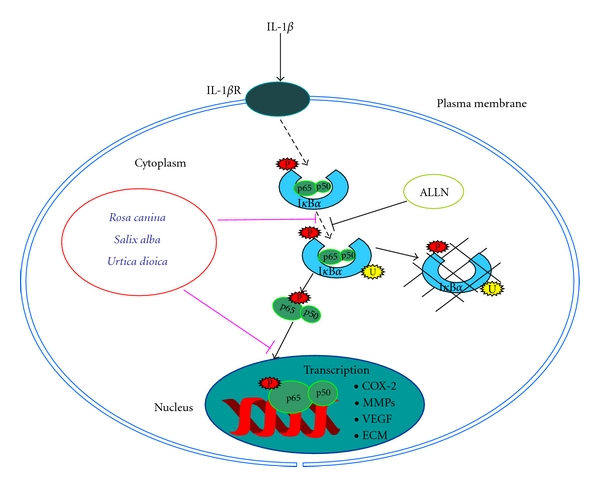
Inhibitory effects of botanical extracts on IL-1*β*-induced NF-*κ*B activation in chondrocytes *in vitro. *IL-1*β* stimulates the IL-1*β* receptor, initiating an intracellular signal transduction cascade, which activates the cytoplasmic I*κ*B*α* kinases (I*κκ*)-*α*, I*κκ*-*β*, and I*κκ*-*γ*. These kinases phosphorylate inactive I*κ*B*α*. Phosphorylated I*κ*B*α* is then ubiquitinated and degraded by the proteasome and active NF-*κ*B is released. NF-*κ*B translocates to the nucleus, where it activates proinflammatory and proapoptotic gene production. In chondrocytes, botanical extracts inhibit the NF-*κ*B signal transduction pathway, ubiquitination of phosphorylated I*κ*B*α* and block translocation of activated NF-*κ*B to the nucleus.

The goal of this study was to characterize the effect and mode of action of three botanical extracts derived from plants with previously reported anti-inflammatory activity on NF-*κ*B expression in primary canine chondrocytes* in vitro*. Under the experimental conditions, (1) botanical extracts inhibited the IL-1*β*-mediated suppression of key extracellular matrix and signaling proteins in chondrocytes; (2) botanical extracts antagonized the IL-1*β*-dependent upregulation of MMP-9, MMP-13, and COX-2; (3) IL-1*β* caused phosphorylation and nuclear translocation of the p65 NF-*κ*B subunit; (4) IL-1*β* caused phosphorylation and subsequent degradation of the inhibitory subunit of NF-*κ*B: I*κ*B*α*; (5) IL-1*β*-induced NF-*κ*B activation and I*κ*B*α* degradation was inhibited by botanicals; (6) finally, in contrast to IL-1*β*-treated cells, the cells treated with botanical extracts redifferentiated into chondrocytes after transfer to high-density culture and produced a cartilage-specific matrix, that is, collagen type II, even when cotreated with IL-1*β*. Therefore, the results obtained strongly suggest that the botanical extracts inhibit IL-1*β*-induced up-regulation of MMP-9, MMP-13, and COX-2 by preventing, at least in part, I*κ*B*α* degradation and NF-*κ*B activation. The schematic in Figure 11 summarizes the possible mode of action of the botanical extracts.

Systematic reviews of clinical studies show little evidence to support botanical remedies as efficacious the treatment for OA [36]. However, the three plants from which extracts have been analyzed in this study have been used in traditional medicines for many centuries and all had claims related to treatment for OA. Whilst patients continue to seek natural remedies to help treat themselves or their companion animals, it is important to provide insights into whether, and if so how, such botanicals may work. As such, *in vitro* models provide an objective benchmark to indicate potential modes of action if translated *in vivo*.

The three botanical extracts in this study are derived from different parts of the plant and previous publications have claimed different active components. The data provided in this study may be used to suggest that there are actives in all three botanicals that can inhibit the IL-1*β*-induced inflammatory process upstream of the I*κ*B*α* phosphorylation step. Whilst the exact mechanism of action remains unknown, the data may also be used to indicate that IL-1*β*-suppressing activities are common to a number of plant species and tissues. With a growing number of publications claiming specific actives, it may be an appropriate time to consider commonalities of botanical extracts rather than focus attention on “unique” attributes of a multitude of specific plants, which may lead to confusion and skepticism toward the area of phytotherapy.

All three botanical extracts had a positive effect on chondrocyte viability, differentiation and function as well as having inhibitory effects on IL-1*β*-induced suppression of proliferation and viability. Furthermore, the three botanicals enhanced mitochondrial activity in chondrocytes, as measured using the MTT assay. Reports have observed botanicals that interfere with the assay through antioxidant (e.g., thiols and flavonoids) reduction of MTT [37], and we have also observed significant negative and insignificant responses with other botanicals (data not shown). Whilst the exact reason for the enhanced mitochondrial activity was not investigated, it was considered an indicator of enhanced metabolism and, as such, was considered a potentially positive attribute.

Cell-matrix interactions in cartilage are essential for the proliferation, differentiation and survival of cells and this interaction is mediated by specific surface receptors, for example, integrins [6, 7, 38]. *β*1-integrins are able to organize cell surface mechanoreceptor complexes [39] and function as signal transduction molecules [40] stimulating MAPkinase pathways [7, 8]. Several studies have already shown that reduced cell-matrix interactions lead to inhibition of Erk1/2 signaling and stimulate the apoptotic pathway in chondrocytes [8]. In this *in vitro* model system, IL-1*β* induced downregulation of collagen type II, CSPG, and integrin expression in chondrocytes. These findings are in agreement with previous *in vitro* studies [18]. Treatment with three botanicals prevented the IL-1*β*-induced inhibition of collagen type II, CSPG, and integrin expression in IL-1*β*-stimulated chondrocytes. 

In this study, IL-1*β* induced upregulation of MMP-9, MMP-13, and COX-2. Cell-matrix interaction requires a permanent remodeling of extracellular matrix proteins executed by MMPs, a group of zinc-dependent endopeptidases that cleave ECM molecules [41] and high levels of MMPs (MMP-1, MMP-3, MMP-9, and MMP-13) are found in the synovium and serum of OA and RA patients [42, 43]. COX-2 is an important mediator of pain and inflammation in OA joints [44] causing PGE_2_ and thromboxane production [45]. PGE_2_ induces many other pathological catabolic effects in cartilage such as decreased proliferation of chondrocytes and inhibition of ECM synthesis [45]. We suggest that the down-regulation of MMPs and COX-2 by botanicals is regulated, at least in part, via NF-*κ*B inhibition, because the expression of these enzymes is regulated by NF-*κ*B [33, 46–48].

Cytokine-induced MMP and COX-2 upregulation is regulated by activation of the ubiquitous transcription factor NF-*κ*B [49]. This transcription factor plays an important role during the pathogenesis of OA, by mediating the expression of catabolic and inflammation-related genes. Interestingly, inhibitors of NF-*κ*B have anti-inflammatory and antidegradative effects in animal models of OA [50]. In the present study, increased phosphorylation of p65 in response to IL-1*β* was demonstrated. This phosphorylation event, in turn, leads to its degradation and subsequent release of activated NF-*κ*B. The results also showed that I*κ*B*α* was completely abolished in the cytoplasmic extracts in chondrocyte cultures treated with IL-1*β* alone, indicating that this cytokine induced its degradation. This indicates NF-*κ*B activation. Treatment of the chondrocyte cultures with the botanical extracts resulted in high concentrations of I*κ*B*α* in the cytoplasm and decreased levels of phosphorylated p65 in nuclear extracts. These results strongly suggest that the botanical extracts inhibit IL-1*β*-induced downregulation of cartilage specific ECM compounds, MAPK-signaling proteins, cartilage-specific transcription factors and upregulation of proinflammatory and degrading enzymes through NF-*κ*B activation by preventing, at least in part, I*κ*B*α* phosphorylation and degradation.

The cartilage-specific transcription factor SOX-9 plays an important role in the expression of cartilage-specific extracellular matrix genes [51]. In this study, a reduction in collagen type II and SOX-9 expression in chondrocytes after treatment with IL-1*β* was observed, in agreement with another study [52]. Other investigators have shown that cytokines partially reduce SOX-9 protein levels through a NF-*κ*B-dependent, posttranscriptional mechanism in mouse chondrocytes [18]. However, by treating cells with the botanical extracts, inhibition of the IL-1*β*-induced NF-*κ*B-dependent downregulation of collagen type II and SOX-9 expression was observed. The results of this study suggest that the botanical extracts markedly suppressed cytokine-induced activation and upregulation of proinflammatory enzymes such as MMPs and COX-2, transcription factor NF-*κ*B and downregulation of cartilage-specific matrix components and important signaling proteins in chondrocytes. Whilst botanical extracts may exhibit multiple modes of action, based on the I*κ*B*α* phosphorylation data, it is probable that inhibition of the IL-1*β* signaling pathway upstream of I*κ*B*α* phosphorylation is likely to be the major cause of the anti-inflammatory activity observed in this study. Monolayer cultures of chondrocytes appear to be a valid model for investigating the mode of action of plant extracts with potential anti-inflammatory properties. However, *in vivo* chondrocytes exist within a three-dimensional extracellular matrix. Therefore, studies were also performed using high-density cultures, which indicated that the botanical extracts do inhibit IL-1*β*-induced inflammation and apoptosis, allowing the cells in high-density cultures to redifferentiate back into chondrocytes. 

## 5. Conclusion

The three botanical extracts used in this study were derived from different parts of the plants used in traditional medicine. Previous publications have claimed different active components and several constituents which have anti-inflammatory effects. In this study similar *in vitro *effects of these three different botanical extracts are important observations and can be used to support the view that they may be potential chondroprotective agents. However, further investigations are needed to characterize the biological entities present within the extracts, to elucidate their subcellular targets *in vitro* and to determine whether they are capable of any similar activity or synergism *in vivo*. 

## Abbreviations

ALLN: Proteasome inhibitor N-Ac-Leu-Leu-norleucinal
AT: Ambient temperature
CSPG: Cartilage-specific proteoglycans
COX-2: Cyclooxygenase-2
DMSO: Dimethyl sulfoxide
ERK 1/2: Extracellular regulated kinases 1 and 2
FCS: Fetal calf serum
IKK: L*κ*B kinase
IL-1*β*: Interleukin-1*β*
MAPK: Mitogen-activated protein kinase
MMP: Matrix metalloproteinase
NF-*κ*B: Nuclear factor-*κ*B
MTT: 3-(4,5-dimethylthiazol-2-yl)-2,5-diphenyltetrazolium bromide
NSAIDs: Nonsteroidal anti-inflammatory drugs
OA: Osteoarthritis; PARP (poly(ADP-Ribose) polymerase)
PBS: Phosphate-buffered saline
Shc: src homology collagen
TIMPS: Tissue inhibitors of MMPs.
